# Analysis of Occludin Trafficking, Demonstrating Continuous Endocytosis, Degradation, Recycling and Biosynthetic Secretory Trafficking

**DOI:** 10.1371/journal.pone.0111176

**Published:** 2014-11-25

**Authors:** Sarah J. Fletcher, Mudassar Iqbal, Sara Jabbari, Dov Stekel, Joshua Z. Rappoport

**Affiliations:** 1 The Centre for Cardiovascular Sciences, Institute of Biomedical Research, University of Birmingham, Birmingham, United Kingdom; 2 School of Biosciences, University of Nottingham, Sutton Bonington Campus, Leicestershire, United Kingdom; 3 School of Mathematics and Institute for Microbiology and Infection, University of Birmingham, Birmingham, United Kingdom; 4 Northwestern University, Feinberg School of Medicine, 303 E. Chicago Avenue, Chicago, Illinois, United States of America; Institut Curie, France

## Abstract

Tight junctions (TJs) link adjacent cells and are critical for maintenance of apical-basolateral polarity in epithelial monolayers. The TJ protein occludin functions in disparate processes, including wound healing and Hepatitis C Virus infection. Little is known about steady-state occludin trafficking into and out of the plasma membrane. Therefore, we determined the mechanisms responsible for occludin turnover in confluent Madin-Darby canine kidney (MDCK) epithelial monolayers. Using various biotin-based trafficking assays we observed continuous and rapid endocytosis of plasma membrane localised occludin (the majority internalised within 30 minutes). By 120 minutes a significant reduction in internalised occludin was observed. Inhibition of lysosomal function attenuated the reduction in occludin signal post-endocytosis and promoted co-localisation with the late endocytic system. Using a similar method we demonstrated that ∼20% of internalised occludin was transported back to the cell surface. Consistent with these findings, significant co-localisation between internalised occludin and recycling endosomal compartments was observed. We then quantified the extent to which occludin synthesis and transport to the plasma membrane contributes to plasma membrane occludin homeostasis, identifying inhibition of protein synthesis led to decreased plasma membrane localised occludin. Significant co-localisation between occludin and the biosynthetic secretory pathway was demonstrated. Thus, under steady-state conditions occludin undergoes turnover via a continuous cycle of endocytosis, recycling and degradation, with degradation compensated for by biosynthetic exocytic trafficking. We developed a mathematical model to describe the endocytosis, recycling and degradation of occludin, utilising experimental data to provide quantitative estimates for the rates of these processes.

## Introduction

Tight Junctions (TJs) provide structural support to epithelial monolayers, regulate paracellular permeability and serve as a barrier to plasma membrane protein diffusion, maintaining apical-basolateral polarity [1],[2]. Numerous human diseases, such as cystic fibrosis and polycystic kidney disease have been demonstrated to involve loss of epithelial polarity [3]. The TJ protein occludin forms homotypic linkages with occludin present in the lateral plasma membrane of adjacent cells [1],[2]. Although occludin is a core member of the TJ complex that links together neighbouring epithelial cells and regulates cell polarity, occludin has also been shown to modulate signal transduction, function as a co-receptor for the Hepatitis C Virus (HCV), and play functional roles during epithelial wound healing [4]–[7]. Our recent work has demonstrated that immediately following monolayer wounding, occludin at the wound edge is rapidly internalised by clathrin-mediated endocytosis within minutes [4]. Other work performed in the same epithelial model has demonstrated that hours following wounding, occludin can be observed at the very leading edge of the migrating front, where it plays an essential role by regulating the localisation of the Par3-aPKC polarity complex [6]. Although occludin is a key regulator of epithelial function and wound healing, the trafficking of occludin has not been well characterised, either in the context of apical-basolateral polarity, or during epithelial wound healing.

Different pathways have been demonstrated to regulate occludin trafficking, potentially based upon the model system analysed [8]–[11]. Additionally, most of the work focusing on the endocytic trafficking of occludin has employed stimuli such as calcium depletion or acute growth factor treatment to induce endocytosis of occludin and loss of epithelial barrier function [9],[11]–[13]. Although there has not been much work performed examining steady-state occludin trafficking, the work that has been performed has been contradictory. Studies by Morimoto et al, 2005 suggest that internalised occludin is predominantly recycled to the cell surface in a Rab13 dependent manner [14], while earlier work indicates occludin is a protein which undergoes rapid lysosomal degradation [15]. Clearly, to further understand the contribution of occludin trafficking to disease pathogenesis, detailed understanding of the mechanisms which regulate occludin localisation at the plasma membrane under basal conditions is required.

We have taken cultured monolayers of Madin-Darby canine kidney (MDCK) cells and employed biotin-based biochemical assays and imaging studies to evaluate occludin trafficking. We specifically focused on trafficking of endogenous occludin during the initial stages of epithelial polarisation in serum-starved steady state conditions. Our results demonstrate that occludin undergoes continuous endocytosis, with the majority of occludin internalised from the plasma membrane within 30 minutes. Following internalisation, intracellular occludin is subsequently lost from detection. We investigated two hypotheses for this loss of intracellular occludin signal: 1) lysosomal degradation, 2) recycling and return to the plasma membrane. Our analyses have demonstrated that this loss of internalised occludin is primarily due to lysosomal degradation, but approximately 20% is returned back to the plasma membrane via recycling endosomes.

Although low level occludin recycling has been demonstrated, a compensatory mechanism must exist in order to replace degraded occludin and maintain occludin homeostasis. Our studies have demonstrated that this loss of occludin is compensated for with newly synthesised occludin which enters the plasma membrane after production. Thus, these results indicate that at steady-state, occludin has only a brief cell surface half-life and that occludin undergoes a constant cycle of endocytic degradative, low-level recycling and biosynthetic secretory trafficking.

Given the experimental data, it is possible to use a dynamical mathematical model coupled with statistical inference techniques to estimate the rates of the molecular processes [16]. We have used such a model to estimate the rates of endocytosis, recycling and degradation of occludin from the experimental data. Moreover, the model predicts that these rates are likely to have slowed considerably as a result of the experimental procedures. However, the rates then recover during the course of the experiment. Thus, together our results represent the first systematic understanding of steady-state occludin trafficking and provide a dynamical mathematical model that will be useful in the understanding of occludin trafficking in polarised cells and during disease processes.

## Experimental

### Cell culture

MDCK (CCL-34 ATCC) epithelial cells were cultured and maintained in DMEM supplemented with 10% Foetal Bovine Serum (FBS), 1% Pen/Strep (both Lonza) in humid conditions at 37°C with 5% CO_2_.

### Cell surface biotinylation

#### Assay 1 - occludin internalisation

MDCK cells were plated into a 6 well plate and 24 hour post-plating the confluent monolayer was serum starved for 1 hour in serum free DMEM in the presence of either DMSO (control) or 250 nM Bafilomycin A (BafA), in humid conditions at 37°C with 5% CO_2_. All reagents were prepared according to the manufacturer's instructions (Pierce). After serum starvation, the cells were washed in ice cold PBS (Lonza) at 4°C. The cells were incubated with Biotin solution at 4°C for 1 hour. Cells were washed in PBS, transferred to serum free DMEM pre-warmed to 37°C containing either 250 nM BafA (Calbiochem) or DMSO (Sigma) incubated in humid conditions at 37°C with 5% CO_2_ for 0, 5, 15, 30, 60 or 120 minutes. After the designated time-points the cells were washed in ice cold PBS, followed by washes with pH 8.6 biotinylation wash buffer (0.5M Tris pH 7.5 (Fisher Scientific), 0.1 M NaCl (Sigma)). Biotin was removed from cell surface proteins by a 30 minute incubation at 4°C with reducing buffer (0.5M Tris pH 7.5, 0.1M NaCl, 15 mM NaOH (Sigma) and 0.1M Sodium 2-mercaptoethanesulfonate (MESNA) (Sigma)). The total amount of plasma membrane localised biotinylated occludin was measured using 2 dishes which were kept on ice post biotinylation, undergoing neither the internalisation period nor cell surface reduction. All dishes were washed in PBS, the cells lysed in 1% Triton-X100 plus Complete Mini-cocktail protease inhibitor (Roche) for 1 hour on ice. Lysates were incubated overnight in spin columns with NeutrAvidin beads (Pierce). Beads were washed in wash buffer plus protease inhibitor, then incubated with 3× Laemmli sample buffer plus 50 mM DTT, for 1 hour and the eluate collected and boiled at 95°C for 15 minutes.

#### Assay 2 - occludin recycling

Cells were biotin labelled as described previously after serum starvation in serum free DMEM. Cells were washed in PBS, transferred to serum free DMEM pre-warmed to 37°C and incubated at 37°C in previously described conditions for a 30 minute internalisation period. Post-internalisation, all but 2 wells underwent biotin removal from cell surface proteins by the previously described reduction step. These wells were then quenched with 20 mM iodacetamide (Sigma) (dissolved in PBS) at 4°C for 30 minutes. Two of these wells were lysed as described previously to enable us to quantify the intracellular biotinylated occludin pool after 30 minutes internalisation. In the two remaining non-reduced wells, cells were quenched as before then lysed to measure total biotinylated occludin (plasma membrane and intracellular biotinylated occludin). The remaining dishes were incubated at 37°C in the previously described conditions for 5, 15, 30 or 60 minutes. After each time-point two dishes were reduced and quenched (to measure the intracellular biotinylated occludin pool) while two dishes were quenched (to measure the total biotinylated occludin pool). Dishes were lysed, the biotinylated proteins were pulled down with NeutrAvidin beads and the proteins eluted from the beads and prepared as described previously.

#### Assay 3 - occludin degradation

All reagents were made up as described previously. MDCK cells were plated into a 6 well plate, 24 hour post-plating cells were washed in PBS, and then incubated for 0, 30, 60, 120 or 240 minutes in serum free DMEM containing either DMSO (control) or 10 µM cycloheximide (CHX). In treatments 0, 30 and 60 minutes the cells were pre-incubated in serum free DMEM, so that in these conditions 2 hours serum starvation occurred before biotinylation. Cell surface proteins were biotin labelled as described previously. Cells were quenched in quenching buffer (Pierce), lysed, incubated with NeutrAvidin, and the biotinylated protein removed from the NeutrAvidin beads and treated as described previously.

### Western blot analysis

Samples from biotin-based recycling assays were run on SDS-PAGE followed by Western blot analysis. For cell surface biotinylation assays 1 and 3, Western Blots were performed as follows: PVDF membranes were probed with rabbit anti-occludin (Invitrogen) primary antibody (1∶500), followed by incubation with goat anti-rabbit IgG horseradish peroxidase conjugated secondary antibody (Thermo Fisher Scientific Inc; 1∶10,000 dilution) and scanned using a CURIX 60 XoGraph machine (AGFA). For cell surface biotinylation assay 2, proteins were transferred onto Odyssey membrane, probed (1∶500 dilution) rabbit anti-occludin (H-279, Santa Cruz Biotechnology) and 1∶10,000 donkey anti-rabbit IRDye 800CW (LI-COR). Western Blots were scanned and the lower band corresponding to occludin was quantified using an Odyssey Infrared Imaging system and software (LI-COR). For film analysis, films were scanned then quantified using NIS Elements. Alterations in antibodies and western blot visualisation technique arose due to an Invitrogen anti-occludin batch difference rendering the antibody incapable of recognising endogenous canine occludin. Thus we validated a Santa-Cruz anti-occludin antibody. This antibody gave high background signal in HRP/Film systems affecting densitometry results; switching to the LI-COR system abrogated background noise.

In both systems, the ROI selection tool a box was drawn around the largest band and the average intensity was measured. This box was used to measure the average band intensity of other bands. Background intensity was measured using the same ROI box moved to a non-band region in the same lane as the band measured. These values were logged to Excel, the average band value was then subtracted from the average background value. For the cell surface biotinylation assay 1 the band intensity value was normalised against the average value for the total plasma membrane biotinylated occludin sample. While in cell surface biotinylation assay 2 the band intensity values for both the total occludin and internalised occludin pools were normalised against the internalised pool value after the initial 30 minutes internalisation period. In the cell surface biotinylation assay 3, the band intensity value was normalised against the time 0 band intensity for either the control or CHX treated cells. The values were obtained from 3 separate experiments and a Student's t-test was performed to determine statistical significance.

### Immunocytochemistry, imaging and co-localisation analysis

MDCK cells were plated onto glass coverslips in 6 well plates. Cells were transfected with CD63-GFP DNA, NPY-mRFP (gifts from Dr Jyoti Jaiswal, George Washington University, Washington D.C., USA) or Rab11-GFP (kindly given by Dr J. Norman, The Beatson Institute of Cancer research, Glasgow, U.K.) using Lipofectamine 2000 (Invitrogen) (4 µg DNA and 20 µl Lipofectamine 2000; incubated as manufacturer recommends). 24 hours post-transfection the confluent monolayer was incubated in DMEM supplemented with 10% FBS, 1% Pen/Strep containing (for experiments examining co-localisation with lysosomal compartments cells were incubated in serum free DMEM containing either 250 nM BafA (Calbiochem) or as a control DMSO for 3 hours in humid conditions at 37°C with 5% CO_2_ prior to fixation). Cells expressing NPY-mRFP, CD63-GFP or Rab11-GFP were washed in PBS (Lonza), fixed in 4% PFA (Electron Microscopy Sciences) and permeabilised with 0.1% Triton-X100 (Sigma). Cells were blocked in PBS plus 10% Normal goat serum (Gibco) and 0.5% BSA (Sigma). Cells were then incubated with mouse anti-occludin (Invitrogen), 1∶100 dilution in block media, for 1 h. Following PBS washes, cells were incubated with goat anti-mouse-AlexaFluor488 (in cells transfected with NPY-mRFP) or donkey anti-mouse-AlexaFluor568 (Invitrogen) (in cells transfected with Rab11-GFP or CD63-GFP), diluted 1∶100 for 1 hour. Post-staining, cells were washed in PBS and mounted onto glass slides using Vectashield with DAPI (Vector Labs). BafA inhibition of lysosomal acidification: MDCK cells were plated into 35 mm MatTek dishes, 24 hours post-plating media was removed and replaced with 2 mls cell culture media plus 75 nM LysoTracker Red DND-99 and either 250 nM BafA or DMSO. The cells were incubated for 2 hours in humid conditions at 37°C with 5% CO_2_. The media was removed and the cells washed in 37°C pre-warmed imaging media (5% FBS in 10 mM HEPES–Hank's balanced salt solution (Sigma) pH 7.4). During imaging cells were maintained at 37°C.

#### Imaging

Cells were imaged using a Nikon A1R inverted confocal microscope using the microscope objective (CFL Plan Apo 60× NA 1.49, Nikon). In all studies a Z-stack with 500 nm increments was performed on each cell imaged, and the images with most intracellular punctate occludin staining selected for analysis. GFP constructs and AlexaFluor488 conjugated secondary antibodies were imaged following excitation with the 488 nm line of an Argon-Ion laser 457–514 nM, mRFP constructs, LysoTracker Red DND-99 and AlexaFluor568 conjugated secondary antibodies were imaged after excitation with Green Diode 561 nm laser. The camera utilised to acquire images was a 12-bit CCD (Ixon 1M EMCCD camera controlled by NIS Elements AR version 3.1/3.2/4).

The system was controlled by Nikon Elements. Analysis of time lapse sequences and still frames was completed using NIS-Elements AR version 3.1, 3.2 or 4 (Nikon). Data was logged into Excel (Microscoft).

### Co-localisation analysis

#### Occludin co-localisation with CD63-GFP

The entire cytosol excluding cell-cell junction staining was selected. Subsequently a Pearson's coefficient comparing the CD63-GFP and occludin signals in the region were obtained. A total of 19 cells were analysed from 3 independent experiments. Pearson's correlation values during control or BafA treatment were compared using a Student's t-test.

#### Occludin co-localisation with Rab11-GFP

Co-localisation analysis for Rab11-GFP was performed by drawing a square ROI within the boundaries of the cell in the occludin image and measuring the Pearson's correlation co-efficient between intracellular occludin and Rab11-GFP structures, to control for random alignment the occludin image was flipped horizontally while the Rab11-GFP image remained in the same position and the Pearson's correlation coefficient repeated. 12 cells from 3 independent experiments were analysed. Control and experiment Pearson's correlation values were analysed for statistical significance using a Student's t-test.

#### Occludin co-localisation with NPY-mRFP

20 intracellular punctate structures per cell in the NPY-mRFP channel were selected using the ROI function and superimposed over the occludin image. The number of NPY-mRFP outlines which co-localise with occludin positive regions were counted. For the control, these regions were moved from NPY-mRFP puncta and again co-localisation with occludin was counted. 13 cells were measured from 3 independent experiments. The number of NPY-mRFP and occludin co-localising vesicle for control and experiment were compared using a Student's t-test.

### Parameter inference methodology

The inference of parameters of the dynamical model was carried out using a Markov Chain Monte Carlo (MCMC) approach, by embedding the mathematical model and experimental data in the inference framework. Specifically, we used Delayed Rejection and Adaptive Metropolis (DRAM) method (described in [17],[18]) and implemented as a MATLAB toolbox (http://helios.fmi.fi/~lainema/mcmc/).

The experimental data under consideration comprise the above experiments quantifying the processes of endocytosis, degradation, and recycling of occludin.

Given the experimental data and parameter values (in the vector *θ*), we define the likelihood function in eq. (1), assuming Gaussian noise. Here **Y** is the data and **Ŷ** is the model output. A sub-routine, calculating the sum of squares term in eq. (1) is fed into the DRAM method.
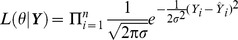
(1.1)The range for parameters ***η_max_*** and ***ρ_max_*** was defined as [0, 10], for ***r*** as [0, 1] while for ***α***, and ***γ*** as [0, 0.5]. Initial proposal covariance matrix was defined as **I**(5)*0.1, where I(5) is a 5×5 identity matrix. With suitable initial values, we ran the DRAM method for 30000 iterations, 2000 burn-ins, and initial σ^2^ as 0.1 with corresponding weight N0 (see [18] for definition) as 10. Adaptation was performed every tenth iteration. All other parameters of DRAM have their default values.

## Results

In order to analyse the potential for endocytosis of occludin during the initial stages of epithelial polarisation, we have made use of a powerful biochemical endocytic trafficking assay [19],[20]. The application of biotin-conjugated NHS esters to cells at 4°C selectively biotinylates lysine residues on the extracellular domains of membrane proteins. When cells are re-incubated at 37°C these membrane proteins can undergo endocytosis and will be transported into the cell. If a biotinylation reagent is used that includes a disulfide bond between the biotin and the NHS group, then biotin linked to proteins remaining in the plasma membrane can be removed by addition of a reducing agent. If the reducing agent is not membrane permeant, then the biotinylated proteins that have been internalised through endocytosis will be “protected” from reduction, and will be retained and available for precipitation with NeutrAvidin beads.

**Figure 1:**
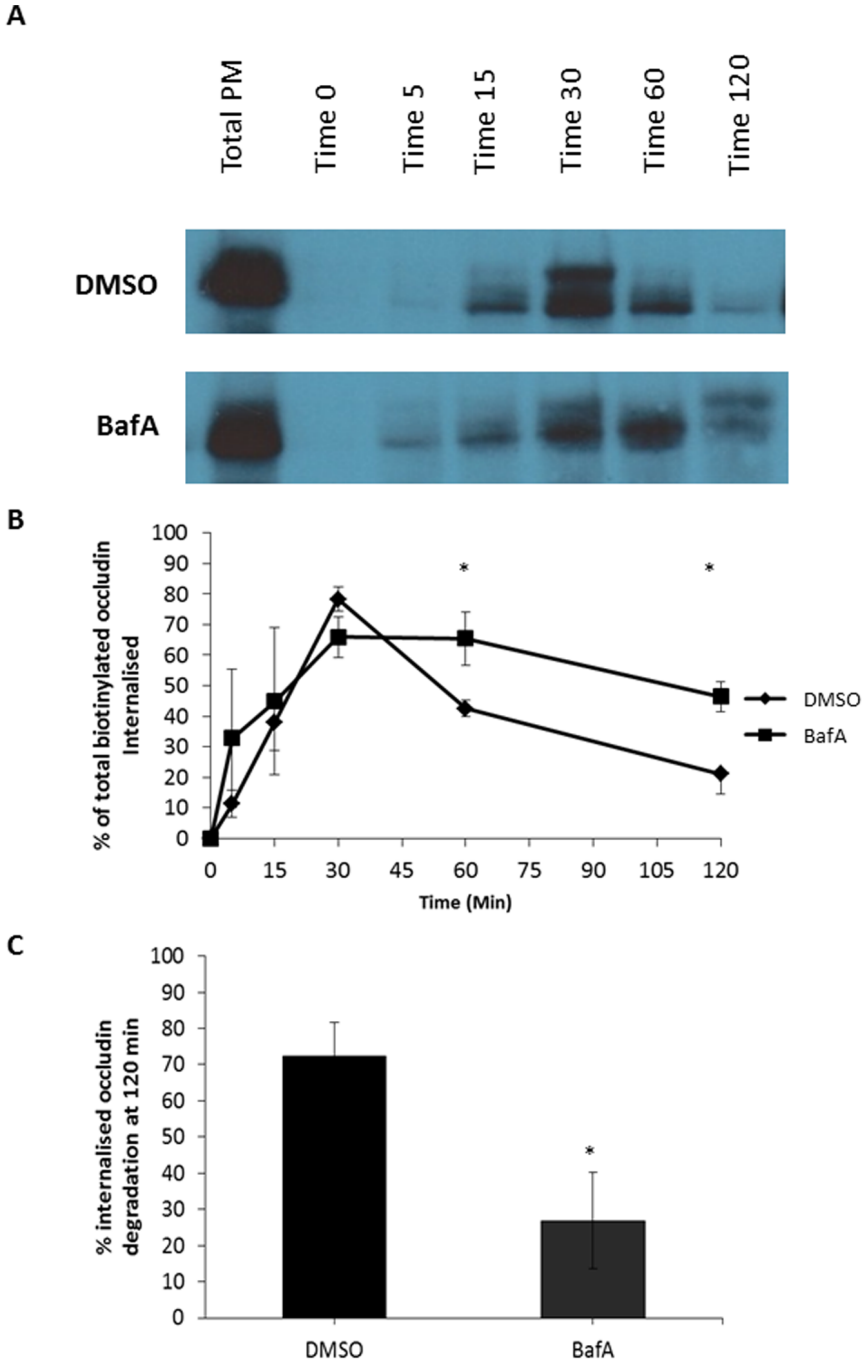
Steady-state endocytosis and degradative trafficking of occludin. A confluent monolayer of MDCK cells were serum starved in the presence of either DMSO or 250 nm BafA. Cell surface proteins were biotinylated and trafficking allowed to recommence for designated time-points in the presence of DMSO or BafA. Cell surface biotinylated proteins were reduced, thus only internalised proteins remain biotinylated. Biotinylated proteins were pulled down with NeutrAvidin beads. Protein abundance was quantified by western blot analysis. (A) Treatment with BafA (inhibitor of lysosomal degradation), attenuates the decrease in occludin signal following endocytosis observed in DMSO treated control cells. (B) Quantification of 3 repeats of the experiments shown in (A). (C) Quantification of the decrease in occludin signal between 30 and 120 minutes comparing control to BafA treatment. * p≤0.05.

As depicted in Figure 1, in confluent monolayers of MDCK cells in culture occludin undergoes continuous steady-state endocytosis. Depicted on a representative blot (Figure 1A) and quantification of 3 experiments (Figure 1B) the internalised intracellular occludin signal rises and subsequently falls. The halftime for internalisation in this study is approximately 15 minutes and nearly all of the occludin present at the plasma membrane undergoes endocytosis within approximately 30 minutes. Extending the time of our analyses beyond this point revealed that the amount of biotinylated occludin present inside the cell post-internalisation subsequently decreased. This decrease in intracellular occludin signal at later time-points indicates a reduction in the level of intracellular occludin following internalisation. There are two hypotheses that could explain the loss of occludin signal subsequent to endocytosis, either the occludin could be recycled back to plasma membrane or undergo degradation.

To determine if the decrease in intracellular occludin signal observed following endocytosis was due to degradation we treated cells with BafA, which inhibits lysosomal acidification and function (Figure S1) [21]. As shown in Figure 1, BafA significantly attenuated the decrease in occludin signal between 30 and 120 minutes approximately 3 fold. Therefore, the majority of occludin loss following endocytosis appears to be due to lysosomal degradation.

**Figure 2:**
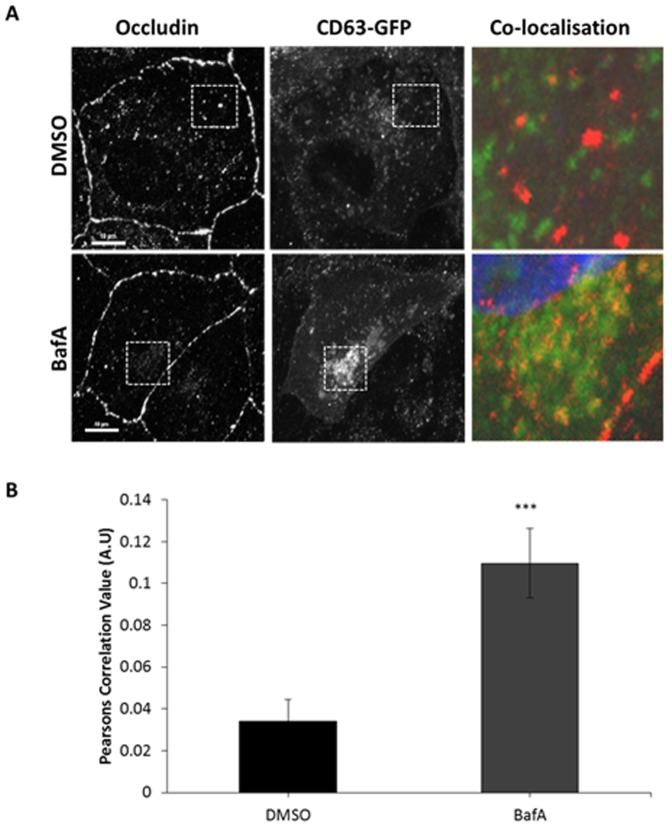
Co-localisation between internalised occludin and late endosome/lysosomal compartments increases after BafA treatment. Incubation with BafA increases co-localisation between occludin and CD63-GFP in comparison to control treated cells. A confluent monolayer of MDCK cells transiently expressing CD63-GFP were incubated with DMSO or 250 nm BafA for 2 hours prior to fixation and immuno-localisation with mouse anti-occludin (1∶100). Cells were imaged by confocal microscopy. (A) Confocal image showing co-localisation between occludin and CD63-GFP in the presence of DMSO or BafA. (B) Quantification of 3 repeats of the experiments shown in (A) with a minimum of 19 cells analysed of each condition. *** p≤0.001.

As a means of further testing this hypothesis, we performed a series of microscopy studies to determine the intracellular localisation of occludin. We employed CD63-GFP (validated by co-localisation with LysoTracker in Figure S2) as a marker of the late endocytic/lysosomal compartment and tested the extent to which occludin was observed to be coincident with CD63 [22]. When immunocytochemistry for occludin was performed in control cells, a very small degree of co-localisation with CD63 was observed (Figure 2A,B). However, the juxtanuclear CD63 positive compartment (in yellow in Figure 2A), was observed to greatly increase with occludin staining following incubation in BafA. Thus, consistent with our above biochemical analyses, BafA treatment resulted in increased co-localisation of occludin to late endosomal/lysosomal compartments (Figure 2A,B). As a very small amount of occludin will be undergoing degradation at any single point, and localisation with degradative compartments could render the protein undetectable by immunostaining, it is not surprising that there is little occludin signal co-localising with CD63 prior to BafA treatment. However, these results confirm our previous observations and demonstrate that occludin undergoes continuous endocytosis and a high level of degradation.

**Figure 3:**
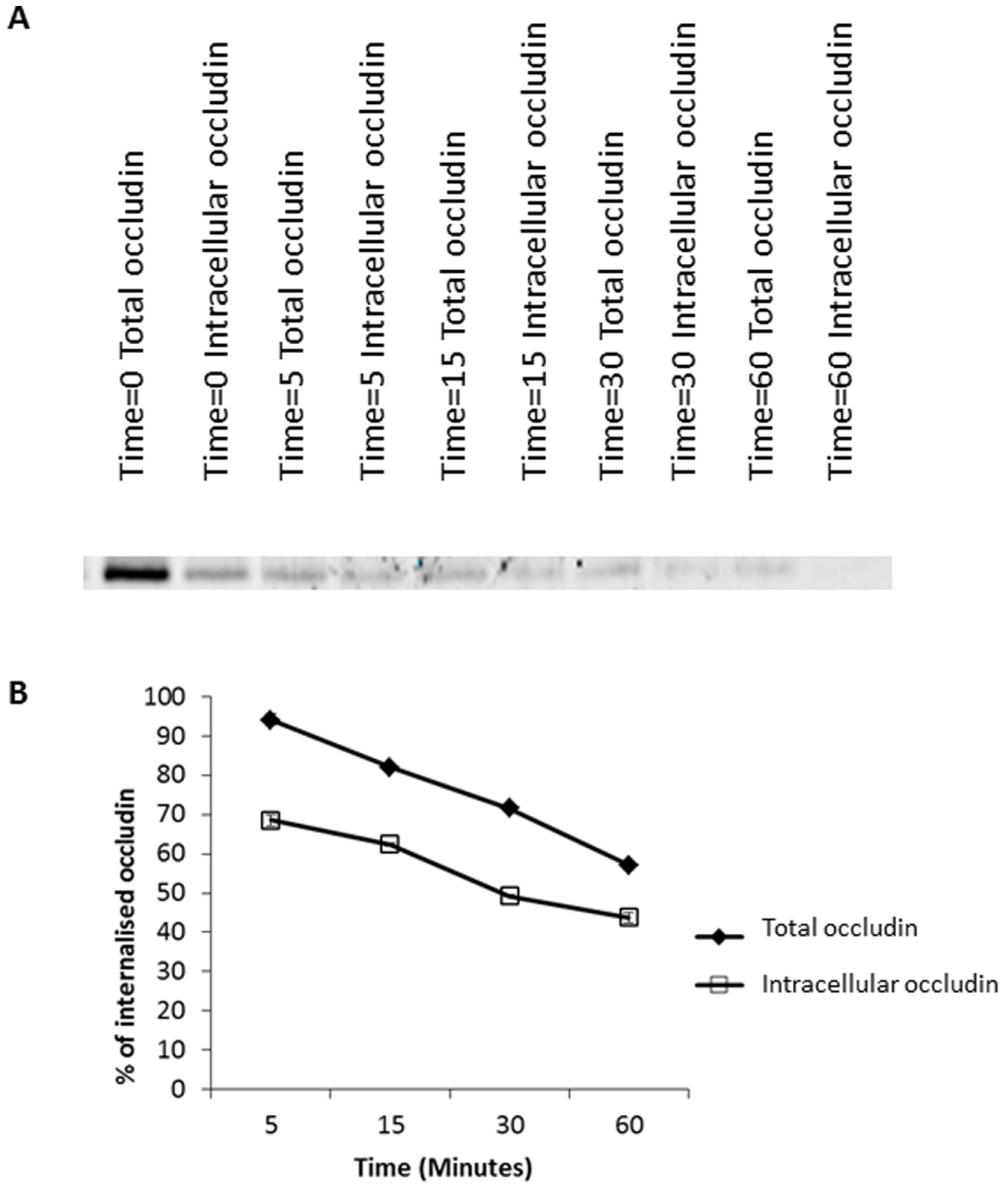
Endocytic occludin recycling. A confluent monolayer of serum starved MDCK cells were biotinylated then warmed to induce occludin internalisation for 30 minutes followed by biotin removal from plasma membrane proteins. Trafficking was allowed to recommence for various timescales and at each time-point the total occludin pool (plasma membrane (PM)+intracellular occludin pool) and the intracellular occludin pool were quantified by additional reduction/quenching steps. Biotinylated proteins were pulled down with NeutrAvidin beads. Occludin abundance was quantified by Western blot analysis. (A) Western blot analysis demonstrating both the total and intracellular occludin pool at various time-points (after a 30 minute internalisation period). (B) Quantification of 3 repeats of the experiments shown in (A) measuring the total occludin levels and the intracellular occludin levels expressed as a percentage of the intracellular pool post-internalisation.

After examining the role of the degradatory pathway in regulation of occludin localisation at the plasma membrane, it was noted that in BafA treated cells there is still a modest reduction in the amount of internalised intracellular occludin from time 30 minutes to 120 minutes, despite inhibition of lysosomal compartment acidification. This could indicate that a small percentage of internalised occludin is being recycled back to the plasma membrane. We investigated this hypothesis using a more complex biotin-based recycling assay which enabled us to quantify the amount of internalised occludin remaining within the cell and the amount returned to the plasma membrane (the intracellular occludin pool subtracted from the total occludin pool) at various time-points after an initial 30 minute internalisation period. As demonstrated in Figure 3, there are higher occludin levels in the total occludin pool samples than in the internalised samples suggesting that post-internalisation occludin is being returned back to the plasma membrane. This was quantified in figure 3B, where occludin levels have been expressed as a percentage of the total amount of occludin internalised (lane Time = 0 Intracellular occludin Figure 3A). Figure 3B demonstrates a steady decrease in both the total occludin pool and the intracellular occludin pool consistent with earlier findings that occludin undergoes a high level of degradation. We also observed a general increase in the ratio of internalised biotinylated occludin to total biotinylated occludin as time increases (Figure S3) with approximately 20% of the internalised occludin being recycled at each time-point.

**Figure 4:**
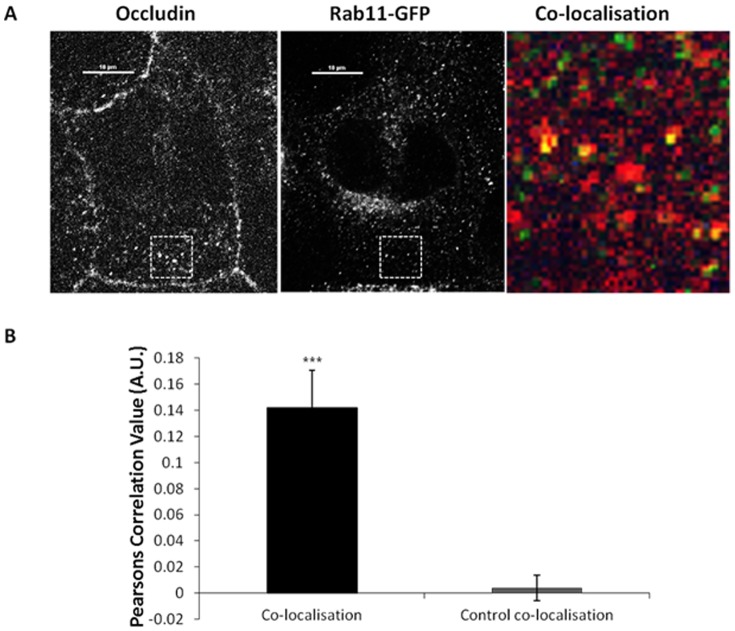
Co-localisation between internalised occludin and recycling endosome markers. A confluent monolayer of MDCK cells transiently expressing Rab11-GFP were fixed and immuno-localisation with mouse anti-occludin (1∶100) was performed. Cells were imaged by confocal microscopy. (A) Confocal image showing co-localisation between occludin and Rab11-GFP. (B) Quantification of 3 repeats of the experiments shown in (A) with a minimum of 12 cells analysed of each condition. *** p≤0.001.

This data has led us to conclude that occludin undergoes low level recycling. We tested this hypothesis further using co-localisation analysis between a marker of the recycling pathway (Rab11-GFP) [23] and endogenous occludin. Co-localisation was identified between exogenous Rab11 and occludin at peripheral punctate structures (Figure 4a), co-localisation analysis demonstrates this level of co-localisation to be statistically significant (Figure 4b). Interestingly, when co-localisation analysis was performed between Rab4-GFP and occludin, no significant level of co-localisation was observed (data not shown). Therefore, this data suggests internalised occludin undergoes recycling back to the plasma membrane via Rab11 positive endosomal compartments alongside degradation.

Our above results suggest that endocytic recycling may only play a minor role in the trafficking of occludin in this system and that a large proportion of occludin is subject to lysosomal degradation, therefore the next question to address was how the occludin at the plasma membrane lost to degradation is being replaced. We tested the hypothesis that the occludin entering the plasma membrane at steady-state to replenish that lost by endocytosis and degradation originates from the biosynthetic pathway. Thus, our next step was to perform cell surface biotinylation to determine the plasma membrane levels of occludin following incubation in the protein synthesis inhibitor CHX [24]. Importantly these were not assays of occludin trafficking directly, but a measure of the amount of occludin present in the plasma membrane after incubation in CHX for different periods of time. This is because if endocytosis and degradation continue and occludin is undergoing constant biosynthetic trafficking into the plasma membrane, CHX treatment will result in an eventual loss of occludin from the plasma membrane.

**Figure 5:**
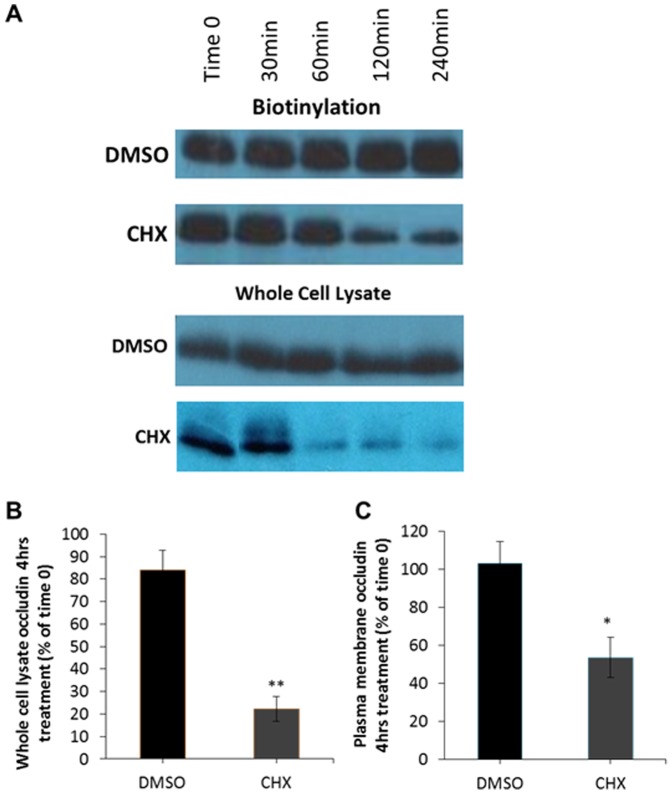
Inhibition of protein synthesis reduces the amount of plasma membrane localised occludin. Serum starved MDCK cells were incubated with serum free DMEM plus DMSO (control) or 10 µM CHX for varying time-points. Cell surface proteins were then biotinylated and pulled down with NeutrAvidin beads. Plasma membrane abundance of occludin was quantified by Western blot analysis. (A) Incubation with CHX reduces both whole cell lysate and plasma membrane occludin levels. (B) Quantification of 3 independent experiments measuring whole cell lysate levels of occludin 4 hours post-CHX treatment normalised to Time 0. (C) Quantification of 3 independent experiments measuring plasma membrane levels of occludin 4 hours post-CHX treatment normalised to Time 0. p≤0.05 and ** p≤0.01.

As demonstrated in Figure 5, following incubation in CHX the amount of occludin observed available for biotinylation in the plasma membrane (Figure 5A,C) and in whole cell lysates (Figure 5B) are both greatly reduced. Quantification demonstrated that CHX treatment for 4 hours reduced the cell surface levels of occludin by approximately 50%, while control cells showed no alteration in the amount of plasma membrane occludin. Therefore, these observations suggest that at serum-starved steady state new occludin trafficked through the biosynthetic system replenishes that lost by endocytosis and degradation.

**Figure 6:**
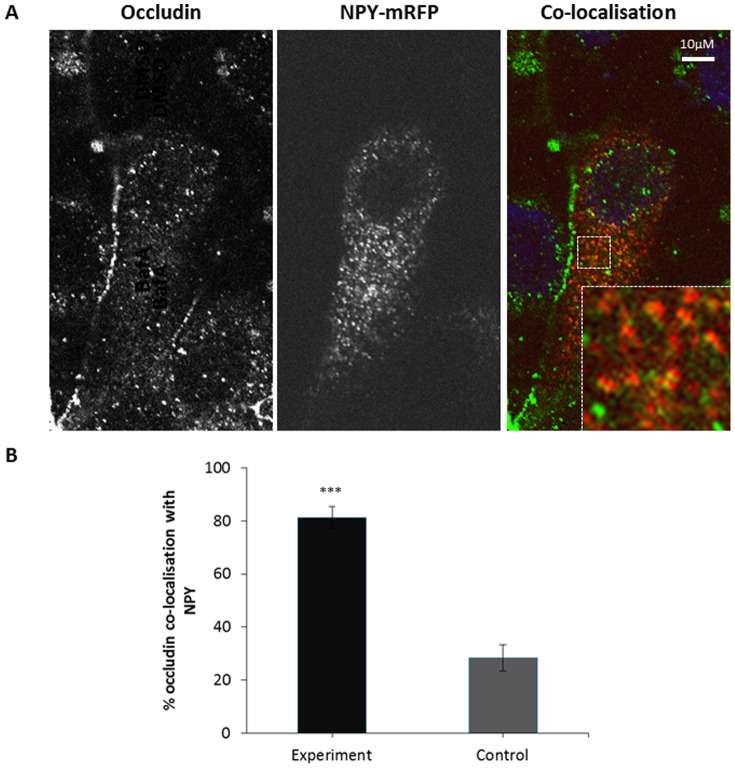
Co-localisation between internalised occludin and markers of the biosynthetic secretory pathway. A confluent monolayer of MDCK cells transiently expressing NPY-mRFP were fixed and immuno-localisation with mouse anti-occludin (1∶100) was performed. Cells were imaged by confocal microscopy. (A) Confocal image showing co-localisation between occludin and NPY-mRFP. (B) Quantification of 3 repeats of the experiments shown in (A) with a minimum of 13 cells analysed of each condition. *** p≤0.001.

In order to further test this hypothesis we evaluated whether occludin co-localises with a marker for the biosynthetic secretory pathway, Neuropeptide Y (NPY) [25]. As depicted in Figure 6 significant co-localisation between occludin and NPY was observed. When controlled for random co-localisation approximately 50% of NPY labelled secretory vesicles were positive for occludin. Thus, this confirms the biosynthetic trafficking of occludin at serum-starved steady state, and taken together these observations demonstrate that occludin undergoes a continuous cycle of biosynthetic exocytic and degradative endocytic trafficking, with a small amount of recycling.

**Figure 7:**
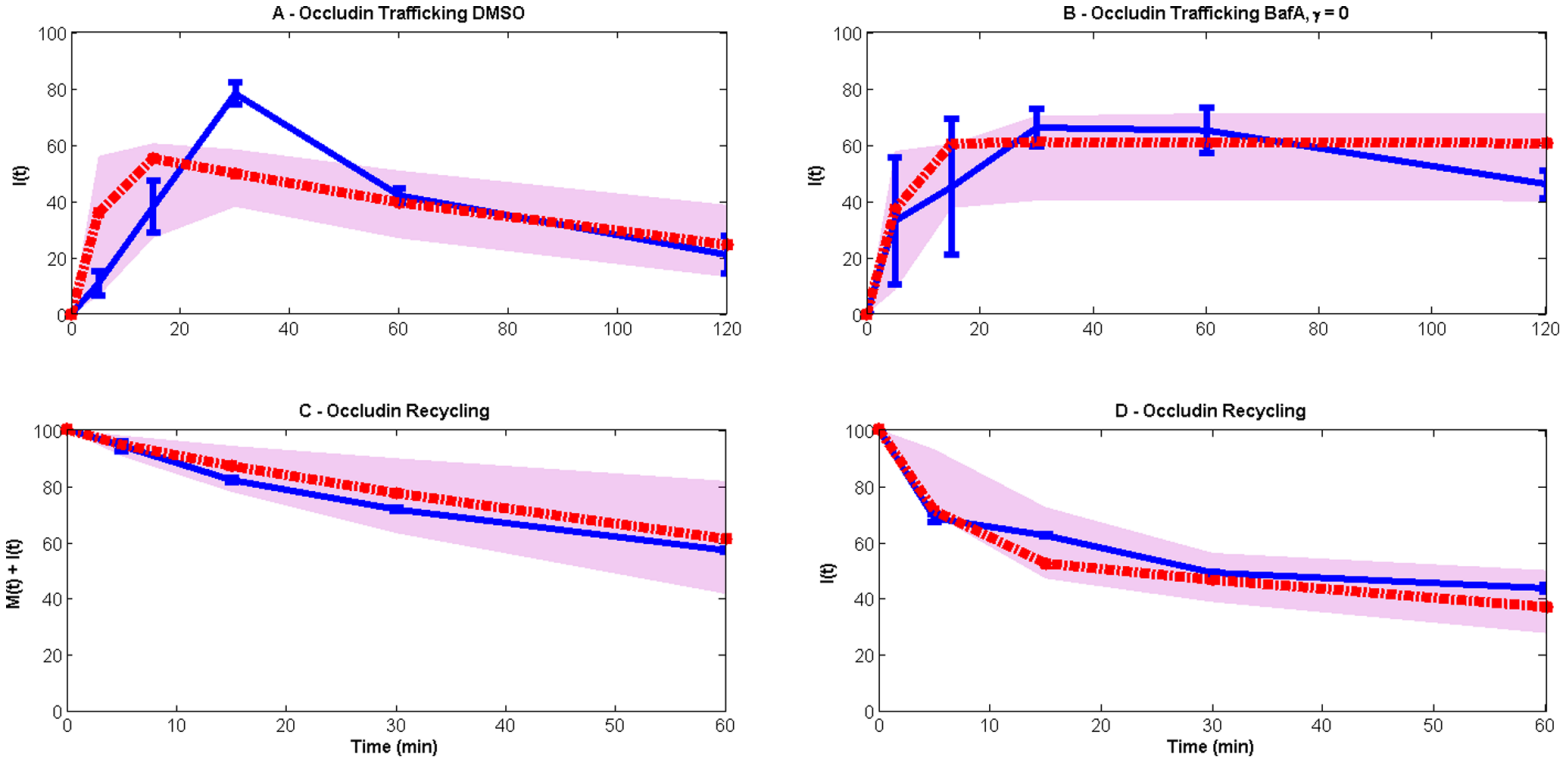
Model (red lines) comparison with experimental data (blue lines). The grey spreads illustrate the model output obtained using 50 random values of parameters from the posterior distribution. The model consists of two variables: *M(t)* (membrane occludin) and *I(t)* (internal occludin). Occludin is assumed to undergo endocytosis, recycling and degradation at rates *η(t), ρ(t)* and *γ* respectively. The resulting equations are *dM/dt = −η(t)M+ρ(t)I, dI/dt = η(t)M−(ρ(t)+γ)I*. *η* and *ρ* are taken to be time-dependent following our finding that a linear version of the model cannot fit the data (Figure 8). We conclude that the experimental procedure that cells be cooled negatively impacts on these processes, which eventually return to maximal rates during the recovery period when cells are warmed. To represent this we use logistic functions: *η(t) = η_max_η_0_e^rt^/(η_max_+η_0_(e^rt^−1))*, and likewise for *ρ(t).* *η_0_* and *ρ_0_* represent the rates of endocytosis and recycling immediately following cooling and are assumed to be a fraction of the maximal rates, i.e. *η_0_ = αη_max_* and *ρ_0_ = αρ_max_* where 0<*α<1* and *r* is the rate of recovery of these processes. (A) and (B) correspond with the data in Figure 1B (steady-state endocytosis and degradative trafficking of occludin) and (C) and (D) to those of Figure 3B (endocytic occludin recycling). In (A) and (B) all occludin is initially on the membrane (*M(0) = 100, I(0) = 0*) and in (B) degradation is inhibited via addition of BafA, hence *γ = 0*. In (C) and (D) all occludin is initially internal (*M(0) = 0, I(0) = 100*). Note that, to correspond with our experimental data, in (C) total occludin is plotted (i.e. membrane and internal occludin), while in (D) only internal occludin is shown. Parameters are estimated to be *η_max_ = 4.64(±2.1)* min^−1^, *ρ_max_ = 3.05(±1.56)* min^−1^, *r = 0.12(±0.15)* min^−1^, *α = 0.02(±0.05)* and *γ = 0.013(±0.002)* min^−1^, where the standard deviations are calculated from the marginal posterior distributions for each parameter.

The mathematical model consists of two equations, one for the membrane-bound occludin (*M*) and one for internal occludin (*I*) (Figure 7). The model includes the processes of endocytosis, recycling and degradation of occludin. The model also includes a slowing of the endocytosis and recycling pathways due to the cooling of the cells as part of the experiment, and the recovery of these processes once the cells have been returned to 37°C.

The model shows an excellent fit to the majority of the data (Figure 7), with only one data point lying outside the region of certainty of the model fits. Thus the model description and parameters are consistent with the experimental data. According to the model fit, the rates of endocytosis and recycling are appreciably impacted by the cooling of the cells, so that at the start of the experiment, the rates of these processes are only 2% of their maximal values. The rates recover to their maximal value with half-life of 32 minutes (as can be calculated from the parameter values given in Figure 7). The maximal rates of endocytosis and recycling both have half-times of under 1 minute – so these are very rapid – although immediately following cooling these are predicted to be slower at 7.5 minutes and 11.4 minutes respectively. The rate of degradation of occludin (assumed to be constant) is predicted to be considerably slower, with a half-time of 53 minutes.

**Figure 8:**
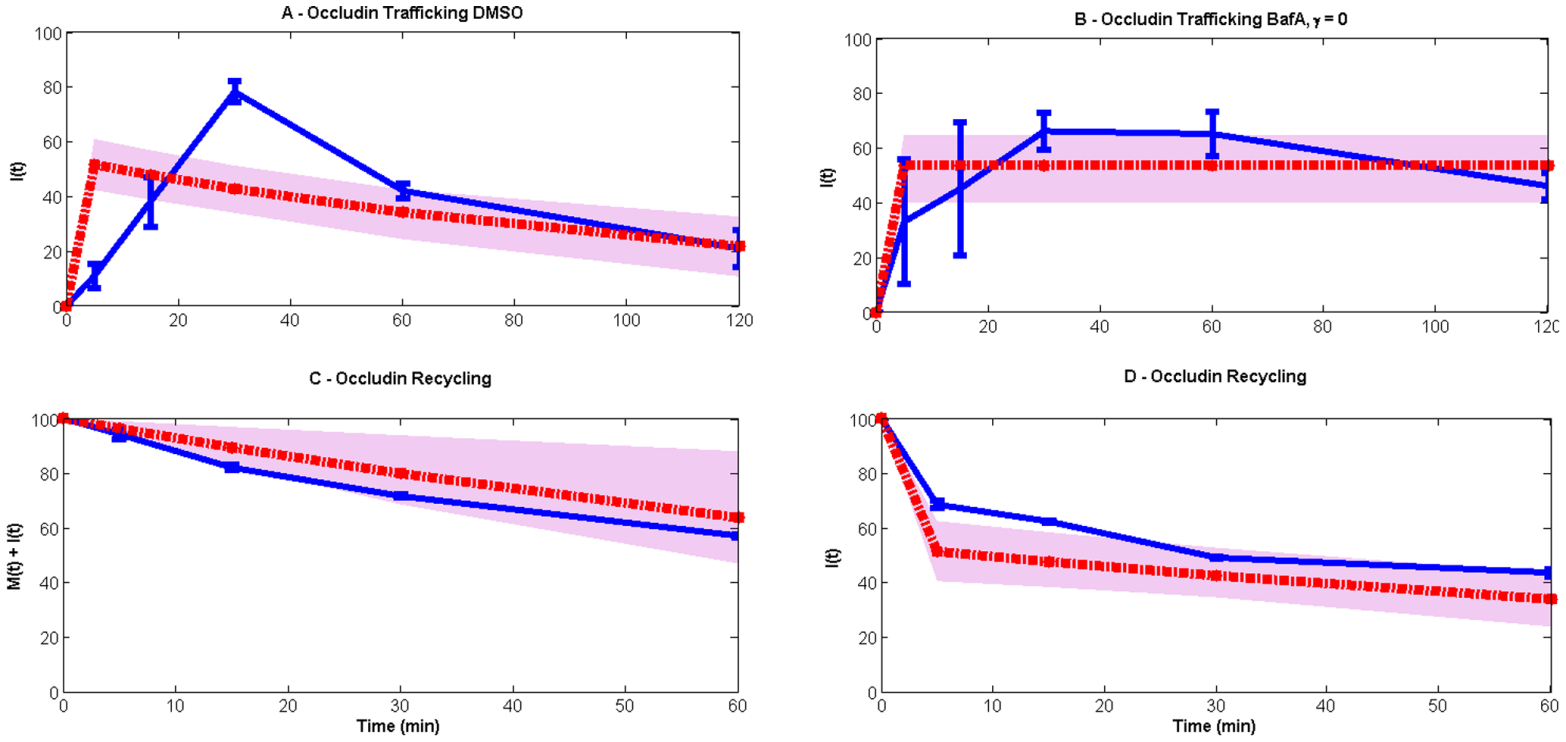
Linear model (red lines) comparison with the experimental data (blue lines). The grey spreads illustrate the model output obtained using 50 random values of parameters from the posterior distribution. (A) and (B) use data taken from the steady-state endocytosis and degradative trafficking of occludin experiment (*M(0) = 100, I(0) = 0*), see Figure 1B, both representing internal occludin with no degradation occurring in (B) (*γ = 0*). Data in (C) and (D) are both obtained from the endocytic occludin recycling experiment (*M(0) = 0, I(0) = 100*), see Figure 3B, the first being the total occludin (*M(t)+I(t)*), and the second internal occludin. The model equations are given by *dM/dt = −ηM+ρI, dI/dt = ηM−(ρ+γ)I*, i.e. the only change from the model in the main text is that the rates of endocytosis and recycling are constant. For all high likelihood parameter values, the initial rise of internalized occludin in the trafficking experiments is considerably faster than experimentally observed. This suggests the necessity of a slower time scale, which is included in the main model as the rate of recovery from cooling.

This model has been compared with a simpler model that does not consider the impact of cooling on the endocytosis and recycling pathways. Such a model is unable to fit the data (Figure 8) because it cannot reconcile the different time scales observed in the experimental data.

## Discussion

TJs are essential to epithelial polarity and together have been implicated in both genetic and infectious diseases [1]–[3],[7]. In particular occludin is emerging as a regulator of numerous physiological and pathological processes both within the TJ complex and through other mechanisms [4]–[7]. Thus, both the steady state and regulated trafficking of occludin and other TJ proteins have been the subject of a great deal of recent interest [9] [11]–[13],[26]–[29]. However, a detailed understanding of the trafficking pathways that regulate occludin localisation has yet to emerge. Thus, we performed a series of biochemical and microscopy studies to determine the mechanisms that regulate the presence of occludin in the plasma membrane of MDCK cells, a very well characterised epithelial model [30]–[32].

First we analysed endocytosis of occludin using a biotinylation based biochemical assay [19],[20]. The results of these studies identified a continuous and rapid endocytosis of occludin at serum-starved steady state. Subsequently the internalised occludin was lost from detection. We then demonstrated through pharmacological inhibition studies that the decrease in occludin signal following endocytosis was due to lysosomal degradation. This hypothesis was confirmed through both biochemical studies, as well as co-localisation analyses with a marker of the late endocytic system, CD63 [22]. This is consistent with evidence from previous studies where occludin has been suggested to undergo post-translational modifications such as ubiquitination and phosphorylation, increasing interactions with degradatory pathway trafficking proteins such as the E3 ubiquitin ligase itch, epsin and epidermal growth factor receptor pathway substrate clone 15 (Eps15) [33].

Although our data indicate that a high level of internalised occludin is degraded, we also investigated the potential for occludin recycling after internalisation. A complex biotin-based recycling assay was employed to examine the role of recycling in regulation of occludin at the plasma membrane. Again we observed a high level of internalised occludin undergoing degradation. We also identified that approximately 20% of internalised occludin was recycled back to the plasma membrane. This was further confirmed in co-localisation studies where we saw a statistically significant co-localisation between the recycling endosome marker Rab11-GFP [23] and endogenous internalised occludin.

Some previous analyses of the continuous constitutive trafficking of TJ proteins have identified a role for endocytic recycling in maintaining plasma membrane levels following endocytosis. However, although both the ESCRT complex and in particular PIKfyve have been implicated in the recycling of claudin, occludin localisation was unaffected by inhibiting these proteins [26],[27]. Interestingly, one study suggested that Rab13 dependant occludin recycling could occur in MTD-1A cells, albeit with a small percentage of total cell surface occludin [28]. Thus, our results are consistent with previous findings suggesting occludin undergoes recycling back to the plasma membrane, however only a small proportion of that which is internalised makes it back to the plasma membrane.

Together, these data demonstrate occludin has a short cell surface half-life and undergoes a continuous process of endocytosis coupled to degradation and low-level recycling during the initial stages of cell polarisation. However, if the majority of internalised occludin is subject to degradation, then it must be replenished through some mechanism.

**Figure 9:**
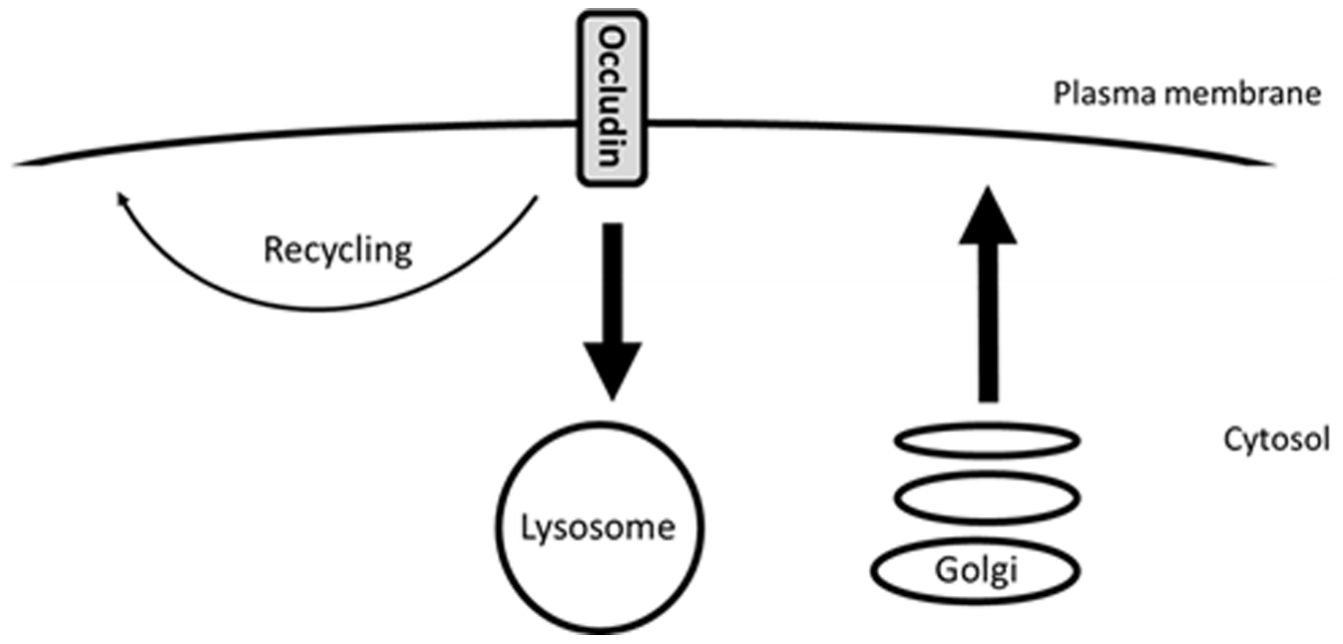
Diagram demonstrating occludin trafficking under steady-state conditions. During the initial stages of MDCK cell polarisation occludin undergoes endocytosis followed by lysosomal degradation and low-level recycling. This is coupled to occludin biosynthesis in order to maintain plasma membrane occludin homeostasis.

Our next step was to determine if biosynthetic trafficking might be responsible for compensating for the loss of plasma membrane occludin due to endocytosis and degradation. We then demonstrated that if we inhibited protein synthesis with CHX and endocytosis and degradation continued, the amount of occludin present in the plasma membrane decreased. Further in support of our conclusion that biosynthetic trafficking accounts for the exocytic replenishment of occludin following endocytosis, we observed significant co-localisation of occludin with the marker of the secretory pathway NPY. Therefore, taken together, our results demonstrate that at serum-starved steady state occludin undergoes a continuous cycle of endocytosis, low-level recycling and degradation which is matched by biosynthetic exocytic trafficking (Figure 9).

The mathematical model has made quantitative predictions for the rates of endocytosis and recycling. These predictions suggest that these rates are very rapid, and that some of the slower time scales observed in the experimental data may be due to a slowing of these pathways due to the cooling regime used to generate the experimental data. This is consistent with previous studies as it is well know that incubation of cells at 4°C inhibits endocytosis [34], and a recovery period to allow trafficking to recommence following re-warming is not at all surprising.

**Figure 10:**
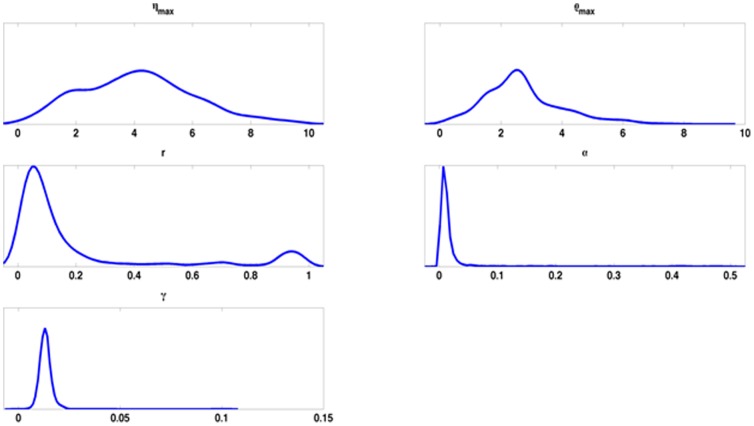
Posterior distributions of the estimated parameters. *γ, α* and *r* display particularly tight distributions around their means, the implication being that their estimates are relatively reliable. *η_max_* and *ρ_max_* (the maximal rates of endocytosis and recycling), on the other hand, display a much broader distribution, meaning that our knowledge of these parameters given the experimental data is less certain.

However, it is also important to take into account the level of uncertainty associated with the parameter estimations. The approach we have taken to model fitting is a Bayesian approach that produces what is known as “posterior distributions” for each parameter (Figure 10). These represent our level of knowledge about the parameter values given the experimental data and the model. The posterior distributions for the occludin degradation rate, the rate of recovery from cold, and the impact of the cold show clearly defined peaks with a tight distribution about optimal values. On the other hand, the distributions for the maximal rates of endocytosis and recycling are considerably broader, indicating less certainty in our knowledge of these rates. Put another way, these distributions indicate that there are other combinations of parameter values for the maximal rates that fit the data almost as well. Better estimates for these rates would require higher resolution time courses for longer periods of time.

There are two data points that the model does not fit especially well. The first of these is the very high level of internalized occludin observed after 30 minutes in the DMSO internalization experiment. This may be due to temperature effects on lysosomal degradative trafficking similar to that observed in endocytosis studies. Previous studies have demonstrated a temperature reduction to 20°C led to decreased lysosomal degradation and accumulation of internalised dextran in late endosomal compartments [35]. These decreased degradation kinetics will be slower in comparison to the prediction of the model. However, degradative trafficking post-endocytosis is complex, involving multiple pathways. Thus incorporation of a recovery period into the degradative trafficking model would not be as straightforward as an endocytosis delay. The second is the lower level of internalized occludin observed after 120 minutes following the addition of BafA. This could be modelled with a partial, rather than total, inhibition of degradation in the presence of BafA. In each case, however, in order to fit these data, the model would require modifications to include extra parameters. It would not be possible to estimate these parameters in a statistically valid way, as each such parameter would depend on only a single data point.

Our detailed mechanistic analysis of occludin trafficking at serum-starved steady state is relevant for many reasons. TJ are critical in the connections between cells and the maintenance of apical basolateral polarity [1],[2]. Although regulated trafficking of TJ proteins has been the subject of several recent investigations, constitutive trafficking has not been as well studied [9],[11]–[13],[36]. Thus, these results are important for two key reasons: serum-starved steady state trafficking is the baseline upon which physiologically relevant alterations and perturbations must be built. Furthermore, occludin has already been shown to function beyond a role in TJ formation [4]–[7]. Therefore, the analysis and modelling of the serum-state steady state trafficking of occludin represents a key step in understanding the roles of this interesting and important protein in numerous physiological and pathological contexts.

## Supporting Information

Figure S1 **BafA treatment inhibits lysosomal acidification and retention of LysoTracker in lysosomal compartments.** A confluent monolayer of MDCK cells were incubated in culture media plus 75 nM LysoTracker Red DND-99 and either 250 nM BafA or DMSO for 2 hours. Live cells were imaged by confocal microscopy. A) Confocal image showing LysoTracker staining in MDCK cells treated with either DMSO or BafA. (B) Quantification of 3 repeats of the experiments shown in (A) with average intensities quantified from a minimum of 20 cells analysed per experiment.
(TIF)

Figure S2 **LysoTracker Red DND-99 co-localises with CD63-GFP.** A confluent monolayer of MDCK cells transiently expressing CD63-GFP were incubated with 75 nM LysoTracker Red DND-99 for 2 hours. Live cells were imaged using confocal microscopy. A high level of co-localisation was observed between CD63-GFP and LysoTracker positive structures (n = 1).
(TIF)

Figure S3 **Increased ratio between internal levels of biotinylated occludin and total levels of biotinylated occludin was observed over time.** A confluent monolayer of serum starved MDCK cells were biotinylated then warmed to induce occludin internalisation for 30 minutes followed by biotin removal from plasma membrane proteins. Trafficking was allowed to recommence for various timescales and at each time-point the total occludin pool (plasma membrane (PM)+intracellular occludin pool) and the intracellular occludin pool were quantified by additional reduction/quenching steps. Biotinylated proteins were pulled down with NeutrAvidin beads. Occludin abundance was quantified by Western blot analysis. Ratio demonstrated the intracellular biotinylated intracellular occludin divided by the total biotinylated occludin levels. Quantification of 3 repeats of experiments.
(TIF)
