# Supplementary figures and images for: Analysis of Occludin Trafficking, Demonstrating Continuous Endocytosis, Degradation, Recycling and Biosynthetic Secretory Trafficking
Source: PLoS One. 2014 Nov 25;9(11):e111176. doi: 10.1371/journal.pone.0111176 (PMC4244031; doi:10.1371/journal.pone.0111176)

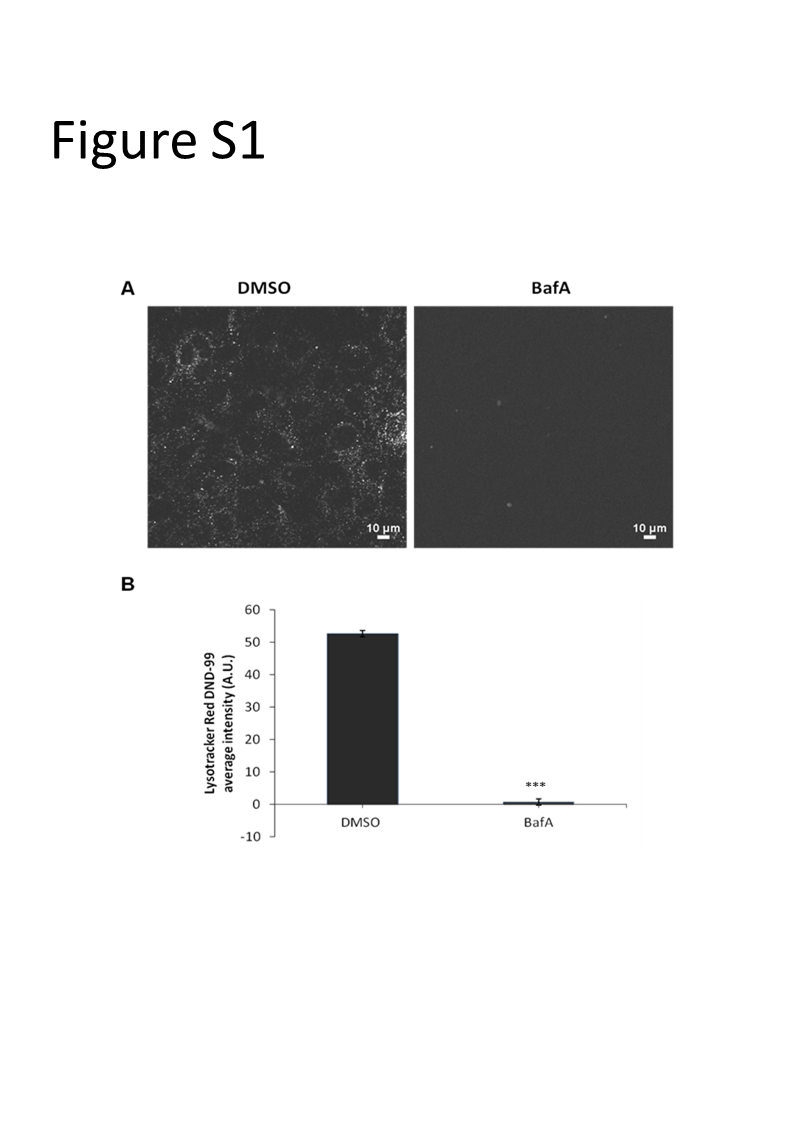

Supplement: Figure S1 — BafA treatment inhibits lysosomal acidification and retention of LysoTracker in lysosomal compartments. A confluent monolayer of MDCK cells were incubated in culture media plus 75 nM LysoTracker Red DND-99 and either 250 nM BafA or DMSO for 2 hours. Live cells were imaged by confocal microscopy. A) Confocal image showing LysoTracker staining in MDCK cells treated with either DMSO or BafA. (B) Quantification of 3 repeats of the experiments shown in (A) with average intensities quantified from a minimum of 20 cells analysed per experiment. (TIF) [file pone.0111176.s001.tif]

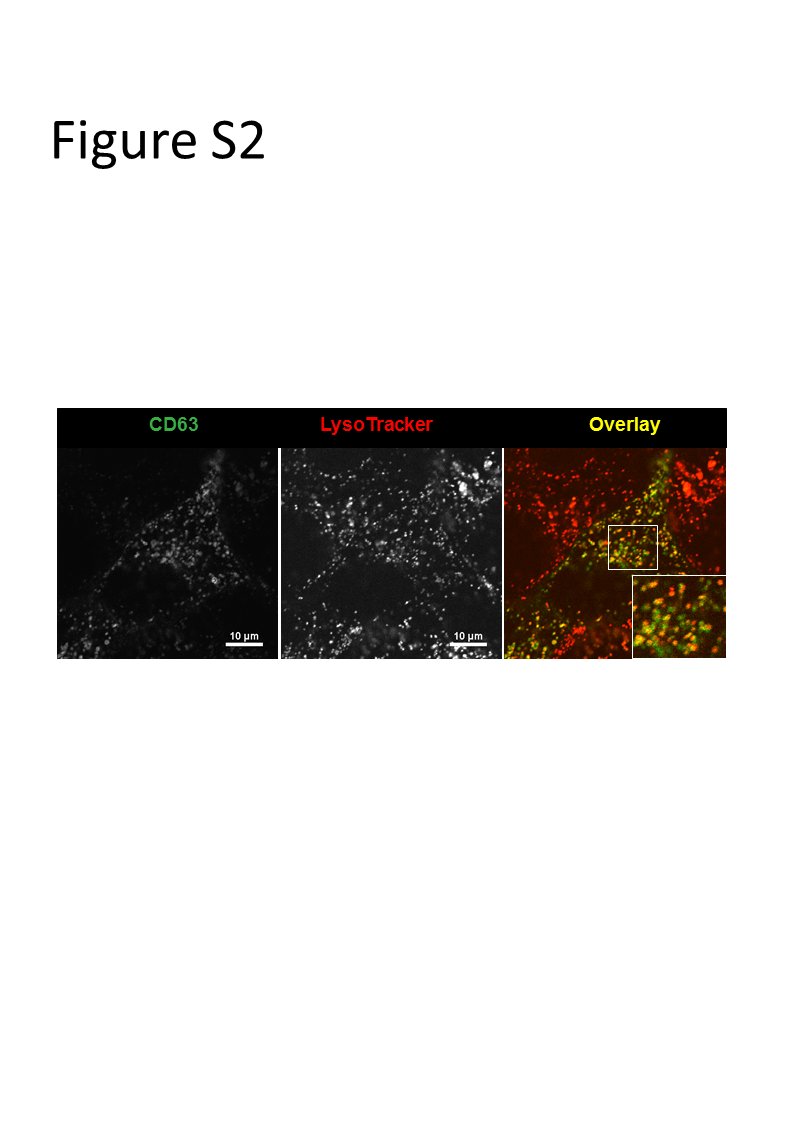

Supplement: Figure S2 — LysoTracker Red DND-99 co-localises with CD63-GFP. A confluent monolayer of MDCK cells transiently expressing CD63-GFP were incubated with 75 nM LysoTracker Red DND-99 for 2 hours. Live cells were imaged using confocal microscopy. A high level of co-localisation was observed between CD63-GFP and LysoTracker positive structures (n = 1). (TIF) [file pone.0111176.s002.tif]
